# Indigenous Peoples' Rights in Data: a contribution toward Indigenous Research Sovereignty

**DOI:** 10.3389/frma.2023.1173805

**Published:** 2023-05-04

**Authors:** Maui Hudson, Stephanie Russo Carroll, Jane Anderson, Darrah Blackwater, Felina M. Cordova-Marks, Jewel Cummins, Dominique David-Chavez, Adam Fernandez, Ibrahim Garba, Danielle Hiraldo, Mary Beth Jäger, Lydia L. Jennings, Andrew Martinez, Rogena Sterling, Jennifer D. Walker, Robyn K. Rowe

**Affiliations:** ^1^Te Kotahi Research Institute, University of Waikato, Hamilton, New Zealand; ^2^Mel and Enid Zuckerman College of Public Health, University of Arizona, Tucson, AZ, United States; ^3^Native Nations Institute, Udall Center for Studies in Public Policy, University of Arizona, Tucson, AZ, United States; ^4^Anthropology and Program in Museum Studies, New York University, New York, NY, United States; ^5^Consultant, Santa Fe, NM, United States; ^6^Forest and Rangeland Stewardship Department, Colorado State University, Fort Collins, CO, United States; ^7^American Indian Center, University of North Carolina at Chapel Hill, Chapel Hill, NC, United States; ^8^Faculty of Health Sciences, McMaster University, Hamilton, ON, Canada; ^9^School of Computing, Queen's University, Kingston, ON, Canada

**Keywords:** Indigenous, Data Sovereignty, data governance, care principles, self-determination, rights

## Abstract

Indigenous Peoples' right to sovereignty forms the foundation for advocacy and actions toward greater Indigenous self-determination and control across a range of domains that impact Indigenous Peoples' communities and cultures. Declarations for sovereignty are rising throughout Indigenous communities and across diverse fields, including Network Sovereignty, Food Sovereignty, Energy Sovereignty, and Data Sovereignty. Indigenous Research Sovereignty draws in the sovereignty discourse of these initiatives to consider their applications to the broader research ecosystem. Our exploration of Indigenous Research Sovereignty, or Indigenous self-determination in the context of research activities, has been focused on the relationship between Indigenous Data Sovereignty and efforts to describe Indigenous Peoples' Rights in data.

## Introduction

Indigenous Peoples' right to sovereignty forms the foundation for advocacy and actions toward greater Indigenous self-determination and control across a range of domains that impact Indigenous Peoples' communities and cultures (Carroll S. C. et al., 2021). Declarations for sovereignty are rising throughout Indigenous communities and across diverse fields, including Network Sovereignty (Duarte, 2017), Food Sovereignty (Blue Bird Jernigan et al., 2021), Energy Sovereignty (Rodon et al., 2021), and Data Sovereignty (Kukutai and Taylor, 2016; Carroll et al., 2019). Indigenous Research Sovereignty draws on the sovereignty discourse of these initiatives to consider their applications to the broader research ecosystem. Our exploration of Indigenous Research Sovereignty, or Indigenous self-determination in the context of research activities, focuses on the relationship between research and Indigenous Data Sovereignty and recent work by the Collaboratory for Indigenous Data Governance and the Global Indigenous Data Alliance (GIDA) to articulate Indigenous Peoples' Rights in data.

The United Nations Declaration on the Rights of Indigenous Peoples (UNDRIP) reaffirms the rights of Indigenous Peoples around the world to access and control data about their Peoples, territories, lifeways, and resources (UNDRIP, Davis, 2016), in Articles 3, 4, 5, 15(i), 18, 19, 20(i), 23, 31, 32, 33, 38, and 42 (Davis, 2016). UNDRIP also recognizes the need to “respect and promote the rights of [I]ndigenous [P]eoples affirmed in treaties, agreements and other constructive arrangements with States” (UNDRIP, Annex). Rooted in Indigenous Peoples' inherent sovereign rights and grounded in UNDRIP, Indigenous Data Sovereignty (IDSov) is concerned with the recognition of Indigenous Peoples' rights and interests in data and enhancing Indigenous Peoples' control of data to which they relate or are linked (Walter et al., 2021). IDSov emerged in the context of increasing digitisation of knowledges and the shift toward open data, which generated ethical discussions about whether data linking initiatives had a social and/or cultural license for data use (Data Futures Partnership, 2017). IDSov expands on the fields of Indigenous Peoples' rights (UNDRIP), Indigenous Cultural Intellectual Property (Mataatua Declaration and UNDRIP art.31), and Indigenous Research Ethics (AIATSIS, 2020; George et al., 2020; Carroll et al., 2022a,b; FNIGC, 2023), synthesizing key arguments and focusing on their application to existing and new forms of data (Carroll S. R. et al., 2021).

IDSov is being operationalized through regional principles like the First Nations' Principles of OCAP^®^, developed by the First Nations Information Governance Center in Canada, which prioritize First Nations' Ownership, Control, Access, and Possession over data related to First Nations (FNIGC, 2023). Similarly, the global CARE Principles for Indigenous Data Governance (Carroll et al., 2019) highlight Collective benefit, Authority to control, Responsibility, and Ethics as core principles when governing Indigenous data (Carroll et al., 2020). Current efforts, in collaboration with the GIDA, national networks, Indigenous Peoples, and allied academics and activists, are developing implementation strategies for the CARE Principles and supporting Indigenous-led data innovations from community data governance to the creation of Indigenous data standards. While principles provide a framework to support ethical decision-making, inform law and policy, and shape infrastructure, they are not legally enforceable. Supporting the effective operationalization of IDSov and Indigenous Data Governance beyond general assertions, necessitates an articulation of what Indigenous rights and interests in data are. Fundamentally, we posit that the shift toward identifying Indigenous data rights and interests could provide the basis for legal recognition, either through Indigenous Peoples own resolutions and codes or national/federal law (e.g., AIATSIS, 2020; Carroll et al., 2022a).

## Indigenous Data Sovereignty and research

The IDSov movement, which emphasizes the rights of Indigenous Peoples to govern the collection, ownership, and application of their own data, has increased in scope and impact since 2015 with a strong focus on research environments. After the publication of *Indigenous Data Sovereignty: Toward an Agenda* (Kukutai and Taylor, 2016), a global network of IDSov national-level networks formed the Research Data Alliance (RDA) International Indigenous Data Sovereignty Interest Group in 2017. The goals of the Interest Group are clearly aligned with the RDA's mission of creating a global community to develop and adopt infrastructure that promotes data-sharing, data-driven research, and data use. Interest Group members are strong advocates for Indigenous-led data-driven research and data use, and are also working in varied ways to build research data capabilities that benefit Indigenous communities. The Interest Group has hosted numerous RDA Plenary sessions, including a workshop at the RDA/International Data Week in Gaborone, Botswana in 2018, which led to the creation of the CARE Principles for Indigenous Data Governance. The CARE Principles were released by the Global Indigenous Data Alliance (GIDA) and published in CODATA's Data Science Journal (Carroll et al., 2020). These principles describe the key outcomes for enhancing Indigenous Peoples' participation in data governance activities. The CARE Principles also highlight critical considerations for non-tribal data actors to recognize Indigenous Peoples' rights and interests in data and calls for greater access to usable data, reflecting the crucial role of data in advancing Indigenous Peoples' innovation and self-determination (Carroll S. R. et al., 2021).

Interest Group members have contributed to chapters in *Indigenous Data Sovereignty and Policy* (Walter et al., 2021) and collaborated with the RDA FAIR Data Maturity Model Working Group to publish on Operationalizing the CARE and FAIR Principles for Indigenous data futures (Carroll S. R. et al., 2021). The CARE Principles appear in the *UNESCO Recommendation on Open Science*, the *AIATSIS Code of Ethics for Aboriginal and Torres Strait Islander Research*, the *Aotearoa New Zealand Antarctic and Southern Ocean Research Directions and Priorities*, and most recently in the Draft decision submitted on Digital sequence information on genetic resources as part of COP15 for the Convention on Biological Diversity. Through the International Work Group for Indigenous Affairs', GIDA members have contributed chapters that explore annual progress in IDSov, released through *The Indigenous World* (Kukutai et al., 2020; Carroll S. R. et al., 2021; Rowe et al., 2022). Scholarly outputs from the Collaboratory for Indigenous Data Governance also include manuscripts that describe how Indigenous Peoples' own laws, policies, and practices set the expectations for research with their peoples, non-human relations, governments, and communities (Carroll et al., 2022a,b).

While IDSov can be applied to data in all forms and contexts, the research ecosystem, as a key generator and user of data, has been a primary site for intervention. Enhancing Indigenous Peoples' control of Indigenous data requires a change in research and data sharing practices across the data lifecycle to ensure opportunities for participation in data governance and downstream activities (Liggins et al., 2021; McAlvay et al., 2021; McCartney et al., 2022). This is particularly important as data diversity, increasing the diversity of available datasets for both AI and genomics, becomes an important issue for creating a data responsible society (Drosou et al., 2017; Hudson et al., 2020; Fatumo et al., 2022). Research attracts significant funds, serves as a key driver of policy, and influences the allocation of resources across society (Kukutai et al., 2021). Inequities arise in each of these domains, which creates a narrative for the progression of Indigenous Research Sovereignty through greater Indigenous involvement via control of Indigenous research agendas, Indigenous research activities, and Indigenous research infrastructures. With these, there will be a significant contribution toward a more diverse, equitable, and inclusive society. As data sharing and data re-use is promoted internationally, research environments are subject to more structured approaches to data management including the use of data management plans, data management strategies, and research data management policies (e.g., Tri-Agency Research Data Management Policy 2021; MBIE Open Research Policy 2022). This creates space for more formal engagement with IDSov principles and the need for more practical tools to guide implementation.

## Indigenous Peoples' Rights in Data

Data innovations are occurring at a rapid pace. There are a range of international and local initiatives to link, centralize, store, visualize, and commercialize data. Given the complex range of data types and applications of data across different domains, these rights involve both (1) data that are born digital (raw data, dis/aggregated data, linked data, and metadata), and (2) non-digital tangible and intangible forms that can be digitized. Consistent with other sovereignty-based movements, the specific nature of rights and interests in data are subject to the expectations of each community and the general principles expressed by IDSov networks globally. For these reasons, the purpose of this paper is to delineate and describe the various rights Indigenous Peoples have expressed in relation to their data and knowledges. The rights in data presented here derive from Indigenous Peoples inherent rights, as collectives, to govern their Peoples, territories, lifeways, cultures, traditions, and resources (Kukutai and Taylor, 2016; Smith, 2016) and as reaffirmed by UNDRIP (United Nations General Assembly, 2007; Davis, 2016) and treaties and agreements with nation-states and others (Carroll et al., 2019). Other instruments also recognize existing rights in relation to data including the Nagoya Protocol on Access and Benefit Sharing (Convention on Biological Diversity).

The rights described here were collated by an interdisciplinary team of Indigenous and settler researchers affiliated with the Collaboratory for Indigenous Data Governance and/or GIDA. The rights were identified through an inductive collaborative working group process led by the Collaboratory for Indigenous Data Governance which reviewed IDSov literature and existing implementation activities. Descriptions were drafted and circulated for comment across the networks associated with GlDA before being released on the GIDA-global website. The rights are aligned with the IDSov themes of Data for Governance and Governance of Data (Smith, 2016). *Data for Governance* primarily relates to the ability for Indigenous communities to access and use data themselves, while *Governance of Data* relates to the ability of Indigenous communities to internally steward and externally influence the use of data (Smith, 2016; Carroll et al., 2019).

## Data for governance

### Right to self-determination: *the ability to organize and control data in relation to a collective identity*

Indigenous research methodologies are grounded in understandings of relational accountability (Wilson, 2008). All data actors should work toward creating a sustainable environment of relational accountability with Indigenous Peoples to advance Indigenous Peoples' research sovereignty and data rights. Working in collaboration with Indigenous Peoples, processes to support collaborative consultation and Free, Prior, and Informed Consent (FPIC) can be developed to create spaces and opportunities for Indigenous communities to organize and govern data (United Nations General Assembly, 2007). This is of high importance as Indigenous communities have been identifying data gaps that need to be closed (FNIGC, 2016). Indigenous Peoples are exercising self-determination efforts to fill these gaps by creating community-driven data collection methods; proving that data collection processes done without Indigenous-led consultation and consent fail the given reality of tribal communities (Rainie et al., 2017). Data actors should understand that Indigenous Peoples' data is governed through the guidance of the Indigenous community via their collective identity (United Nations General Assembly, 2007; AIATSIS, 2020; Fernández-Llamazares et al., 2021). Indigenous Peoples' rights to self-determination means that they have the right to amend or change any of the rights herein, in line with evolutions in Knowledge, technology, data, and other advancements, as is necessary.

### Right to reclaim: *the right to reclaim, retain, and preserve data, data labels, and data outputs that reflect Indigenous Peoples' identities, cultures, and relationships*

The reclamation of data includes Indigenous Peoples right to recover and/or repossess known/unknown data about their peoples, communities, ancestors, and non-human relations. The process of reclamation may include Indigenous-led restructuring, reinterpreting, and relearning how data are prioritized, collected, and used in ways that center relational accountability and restores spiritual relationships to non-human relations and to one another. It is important to note that understandings and interpretations may vary by Indigenous Peoples and their traditional governance structures. This may require modifications to state-imposed legal frameworks for data sharing. The reclamation of data and knowledge has also been referred to as repatriation and rematriation. Reclamation supports self-determination and recentring of Indigenous concepts of being and belonging.

### Right to possess: *the ability to exercise jurisdictional control over the ways that data flow/move/are queried*

The right to possess refers to the ability of an Indigenous community to bring data collected about them into their legal control/scope of governance to align data with Indigenous Peoples' values, interests, and aspirations. This legal control/scope of governance can be based Indigenous data being located within Indigenous territory but can also mean that tribal data is amenable to Indigenous governance even if located outside the community's territory through mechanisms like Data Governance Agreements and Future Acquisition Agreements (see FNIGC, 2020, p. 47). This legal basis flips the script on traditional exploitative practices by western institutions gathering and using Indigenous data to advance their own agendas, further harming Indigenous communities along the way (Emanuel and Bird, 2022).

### Right to use: *the ability of individuals and collectives to use data for their own purposes*

Indigenous Peoples and Indigenous or Indigenous-determined data actors have a right of access to use data in a timely way to enable the wellbeing and governance of Indigenous collectives. The right to use includes tribal access to log-in details, tools and devices, and accessible materials in a legible and attainable format. It also includes the provision of necessary skills training for Indigenous Nations on how to access and use tribal data. Effective data use must ensure that Indigenous data are in a language and format accessible to inform Indigenous governance. This includes the rights of Indigenous Peoples to use data for Indigenous-led commercialization purposes and other means that benefit Indigenous communities, on their own terms.

### Right to consent: *the expression of digital autonomy and the ability to assess risks and accept potential harms*

Consent is an ongoing relationship that any party may withdraw from at any time. This right refers to the collective consent of a relevant Indigenous community, who has a relationship, link, or connection to the data. Agreement on the scope, parameters, and limitations of consent should precede any conveyance of consent. In alignment with UNDRIP, Indigenous Peoples have a right to full disclosure, via FPIC, of present and future risks, harms, and benefits before giving collective consent. Indigenous communities have a right to consent to a part but not the whole of a project. There is a responsibility to respect Indigenous Peoples' decisions to give or withhold consent in full or in part.

### Right to refuse: *the right to say “no” to certain uses of data*

The right to refuse is part of the internationally recognized principle of FPIC as described in the “right to self-determination”. The right to refuse encompasses the right of Indigenous Peoples to refuse to release, share, or disclose their data and may be expressed in the context of consent, governance, or privacy. Indigenous communities can also refuse to allow the unauthorized use of their data by outsiders. The right to refuse is a dynamic and ongoing right, which means that Indigenous communities may choose to withdraw previously given permissions and/or refuse to participate in consent or governance processes.

## Governance of data

### Right to govern: *the right to lead and collaborate in the development and implementation of protocols and in decisions about access to data*

Indigenous Peoples should have the right to exercise independent governance over data in a way that supports their communities' visions and priorities. This upholds their right to self-determination, reduces potential future harms, and allows for the development of new data management policies that align with Indigenous communities' goals and values. Building opportunities for community-led evaluation and reporting into data management plans and project timelines will allow for greater understanding of potential concerns and benefits. These actions could support current and future research projects and better inform funders of where to direct their money. Providing information in non-specialized language will support Indigenous communities to [re]build or expand capacities around data governance.

### Right to define: *the right to define lifeways of knowing and being including how they are represented in data*

The right to define includes the ability of Indigenous Peoples' to revise, as needed, the definitions, conceptualizations, and representation of their lifeways and epistemologies (ways of knowing) over time, including the right to define membership and citizenship. As there are many different voices in tribal communities it is important that the right to define takes into account different constituents and perspectives within the Indigenous community. The right to define includes having data used in ways relevant to their worldviews and not contrary to their cultural practices.

### Right to privacy: *the protection of collective identities and interests from undue attention, also including the possibility of requesting omission and/or erasure*

Privacy is an inherent right to Indigenous Peoples and communities (Kukutai et al., 2023). The intent of privacy, in the Indigenous context, is to maintain social interaction and cohesion between individuals and across communities. It includes the ability to protect collective identities from harm and determine when and how the use of data interferes with relationships in these communities. Collective privacy may be maintained through the omission of particular pieces of information within datasets. There may also be times when erasure of data is necessary and appropriate, to maintain the dignity of Indigenous communities.

### Right to know: *the ability to track the storage, use, and reuse of the data and who has had access to them*

Indigenous Peoples' data have been and continue to be extracted and stored in repositories outside of their scope of governance and control. Indigenous communities have the right to know what data has been collected, where it is being stored, and how it is being used. Data collecting agencies should resource processes that enable Indigenous communities to access and review Indigenous collections and data. Increasing visibility and transparency around the use of Indigenous data, why it is needed, and how its use benefits Indigenous communities, increases trust and accountability. The right to know also extends to how data is processed and represented in published materials, presentations, and other media.

### Right to association: *the recognition of provenance and terms of attribution*

Data should be stored with appropriate provenance metadata (data about data and its origin) that support access and governance by Indigenous communities (Golan et al., 2022). Data actors should disclose if data will be made available for future use, by whom, and where it will be located. Metadata should identify appropriate provenance and describe how this will be attributed throughout the entire data lifecycle including secondary use and publication.

### Right to benefit: *the opportunity to benefit from the use of data and equitable benefit sharing from derivatives of data*

Relationships between data actors and Indigenous Peoples have been largely extractive, resulting in harms through data misuse and the continued misappropriation of Traditional Knowledge and Indigenous data (David-Chavez and Gavin, 2018; Emanuel and Bird, 2022). Indigenous communities have a right to benefit from their cultural intellectual property and from the use of their data. Data actors should work with Indigenous Peoples to establish appropriate data sharing frameworks that ensure benefits generated from the use of Indigenous data are subject to fair and equitable benefit sharing.

## Discussion

The rights described above reflect rights, interests, and expectations that have been articulated by Indigenous Peoples in a variety of local, regional, national, and international contexts. Extractive data practices have created data harms, a plethora of data that do not serve Indigenous community needs, and a dearth of data that relate to Indigenous Peoples own values and conceptions of wellbeing. Through consultation and by establishing data governance mechanisms, data actors and Indigenous communities can now define how and when data can be utilized to the benefit of the communities concerned. Indigenous communities should define these benefits as an exercise of strengthening their sovereignty, self-determination, and the health of current and future generations.

While a number of the rights, such as consent, benefit, and privacy, are generally used within data governance frameworks, there are unique dimensions to these rights that reflect the collective nature of many Indigenous worldviews. Other rights, like the right to define, right to association, and the right to reclaim, reflect the desire of Indigenous Peoples to transform settler-colonial frameworks that shape their contemporary experiences. It is clear that not all rights can be met or expressed in every context but the range of rights provides the potential to recognize one or more Indigenous data rights in any context. The onus is on data users to know that these rights exist. Whether or not the community has the capacity to fully exercise those right, the data users should acknowledge and make space for those rights to be realized throughout the process.

Different rights will always be balanced against each other and Indigenous Peoples' rights to data can be understood in the context of three dynamic and interlinked relationships. Ownership and control are implicitly linked to discussions about rights and interests. While there are jurisdictional differences in terms of the application of Intellectual Property (IP) rights to data, the IP framework affects the way in which people shape their thinking about data rights in an ownership/property context rather than a framework of relationships and control. Access and use are shaped by understandings of the exclusive or shared nature of data being managed, which might relate to jurisdictional mandates, commercial sensitivities, cultural imperatives, or legislative requirements. It is also important to consider the relationship between individual consent and collective governance when navigating issues of data sharing, as consent often creates limits around which data governance frameworks can be applied.

The shift toward open data is supported by the growth of data infrastructures, the creation of data standards, the formalizing of data governance policies, and the establishment of data access protocols. It is important that Indigenous Peoples' Rights in Data are considered as these activities evolve into the future. For example, the IEEE is developing a Recommended Practice for the Provenance of Indigenous Peoples Data (SSIT/SC, 2023). This will be the first International Indigenous Data Standard but likely followed by others. On a similar note, International harmonization of data policies is apparent across the research sector with both the promotion of Creative Commons Licenses and recommended use of data management plans. In Canada, formal Research Data Strategies are now mandatory at an institutional level in addition to Data Management Plans for research projects (Innovation Government of Canada, 2022).

**Figure 1:**
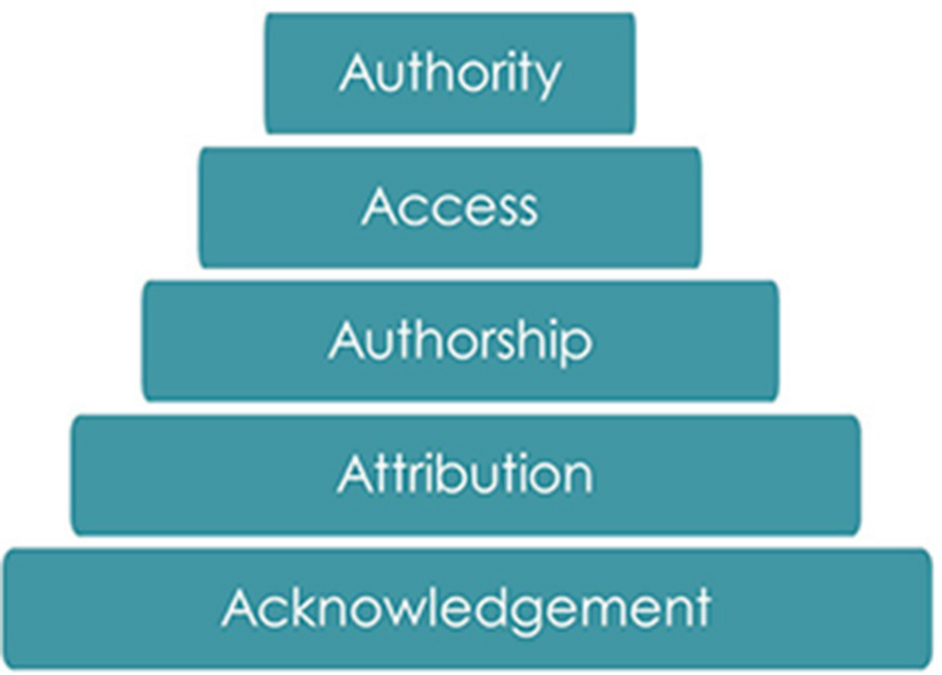
Actions that support and temporally build recognition of indigenous contributions.

We expect the Indigenous Peoples' Rights in Data to be adopted by organizations in a similar way to a consumer code of rights. For any given context for data use or data sharing, the organization can consider which rights they can recognize with Indigenous Peoples as rightsholders. Similarly, Indigenous communities can approach the organization to ask how their rights are being expressed in practice. Action is fundamental to progress, whether that be for recognizing Indigenous principles, like OCAP^®^ and CARE, or the Indigenous Peoples' Rights in Data (Mulder et al., 2022). It may take time to identify mechanisms to give effect to each of the rights. However, that shouldn't limit efforts to engage directly with Indigenous communities and determine what appropriate actions look like across a spectrum, which could include discussions about acknowledgment, attribution, authorship, access, and authority (Figure 1).

Land Acknowledgments are becoming a common practice across US and have a 20 year history in Canada and Australia (McKenna, 2014; Âpihtawikosisân, 2016; Garcia, 2018; Robinson et al., 2019; George, 2022). Guidelines for assessing the efficacy of land acknowledgments in the US are just beginning (Johnson et al., 2023). Acknowledgments of Indigenous communities are often seen within research reports but less frequently within published articles (Lock M. et al., 2022). Specific attributions to Indigenous communities for content (Indigenous knowledges) and/or consent/permits represent a stronger recognition of their contributions to research (Liboiron, 2021). One tangible example is the Attribution Label, part of the suite of Traditional Knowledge Labels developed by Local Contexts, where communities inform anyone who uses the material about who the correct sources, custodians, or owners are (Local Contexts - Grounding Indigenous Rights, 2023). Authorship provides researchers with a way to maintain their link to published material and anywhere it is used through established citation practices. This citation practice lives much longer than the legal rights that copyright might bestow upon an author (Anderson and Christen, 2019; Anderson and Francis, 2021). In response to Indigenous concerns about lack of recognition, a number of journals now require articles with Indigenous content to include Indigenous authors (Cooke et al., 2021; Lock M. J. et al., 2022). Concerns about lack of acknowledgment and attribution around the secondary use of data within open data environment has prompted discussions about data provenance and data access. Increasingly, Indigenous communities are looking at Data Access Agreements to be put in place to address cultural sensitivities and clarify data sharing protocols in a similar manner to projects with commercially sensitive data. Maintaining cultural authority in decision-making processes throughout research projects and long-term data outcomes in relation to any derivatives or secondary activities resulting from research, represents Indigenous communities asserting their self-determination.

## Conclusion

Indigenous concerns about misappropriation of traditional knowledge and Indigenous data grow as the research environment promotes data diversity, facilitates data centralization, encourages data sharing, enables data linkage, and generates pathways that enable the commercialization of data. As the research data environment is increasingly oriented toward open access there is a need to ensure that data systems and practices operate in a manner consistent with the Indigenous aspirations for data sovereignty and research sovereignty. Indigenous Data Sovereignty provides a platform for defining Indigenous narratives and enabling Indigenous research agendas as a tangible expression of Indigenous Research Sovereignty. Establishing Indigenous Peoples' Rights in Data provides a concrete step toward operationalizing Indigenous Data Sovereignty and Indigenous Research Sovereignty by articulating a range of specific rights that can be recognized to support Indigenous Peoples' aspirations for control of data and self-determined research activities.

## Data availability statement

The original contributions presented in the study are included in the article/supplementary material, further inquiries can be directed to the corresponding author.

## Author contributions

MH drafted the initial manuscript based on work developed by all the authors. SC and RR integrated comments. RS and MH finalized the manuscript. All authors contributed to review and edits.
